# Impact of timing of surgery in elderly hip fracture patients: a systematic review and meta-analysis

**DOI:** 10.1038/s41598-018-32098-7

**Published:** 2018-09-17

**Authors:** Thomas Klestil, Christoph Röder, Christoph Stotter, Birgit Winkler, Stefan Nehrer, Martin Lutz, Irma Klerings, Gernot Wagner, Gerald Gartlehner, Barbara Nussbaumer-Streit

**Affiliations:** 1 Danube University Krems, Faculty of Health and Medicine, Department for Health Sciences and Biomedicine, Center for Medical Specialisations, Dr. Karl-Dorrek-Str. 30, A-3500 Krems, Austria; 2 LK Baden-Mödling-Hainburg, Department of Orthopedics and Traumatology, Waltersdorferstraße 75, A-2500 Baden, Austria; 3 Danube University Krems, Faculty of Health and Medicine, Department for Health Sciences and Biomedicine, Center for Regenerative Medicine and Orthopedics, Dr. Karl-Dorrek-Str. 30, A-3500 Krems, Austria; 4 UK Krems, Department of Orthopedic Surgery, Mitterweg 10, A-3500 Krems, Austria; 5 Landeskrankenhaus Hall, Department of Orthopedics and Traumatology, Milser Straße 10, A-6060 Hall in Tirol, Austria; 6 Danube University Krems, Department of Evidence-based Medicine and Clinical Epidemiology, Dr. Karl-Dorrek-Str. 30, A-3500 Krems, Austria; 7 Cochrane Austria, Danube University Krems, Dr. Karl-Dorrek-Str. 30, A-3500 Krems, Austria; 8 RTI International, 3040 Cornwallis Road, Research Triangle Park, North Carolina, NC 27790 United States

## Abstract

We aimed to assess the impact of timing of surgery in elderly patients with acute hip fracture on morbidity and mortality. We systematically searched MEDLINE, the Cochrane Library, Embase, PubMed, and trial registries from 01/1997 to 05/2017, as well as reference lists of relevant reviews, archives of orthopaedic conferences, and contacted experts. Eligible studies had to be randomised controlled trials (RCTs) or prospective cohort studies, including patients 60 years or older with acute hip fracture. Two authors independently assessed study eligibility, abstracted data, and critically appraised study quality. We conducted meta-analyses using the generic inverse variance model. We included 28 prospective observational studies reporting data of 31,242 patients. Patients operated on within 48 hours had a 20% lower risk of dying within 12 months (risk ratio (RR) 0.80, 95% confidence interval (CI) 0.66–0.97). No statistical significant different mortality risk was observed when comparing patients operated on within or after 24 hours (RR 0.82, 95% CI 0.67–1.01). Adjusted data demonstrated fewer complications (8% vs. 17%) in patients who had early surgery, and increasing risk for pressure ulcers with increased time of delay in another study. Early hip surgery within 48 hours was associated with lower mortality risk and fewer perioperative complications.

## Introduction

Hip fractures in elderly populations are a major public health concern in Europe and the United States (US)^1–3^. The annual incidence of hip fractures rises with age. In the US, it ranges between 0.2% in women aged 60 to 64 years to 2.5% in women aged 85 years or older^4^. In Europe, the annual hip fracture incidence for elderly women aged 60 years or older ranges between 0.5% to 1.6% per year^5–7^. The risk for men is about half of that for women^8^.

Hip fractures in elderly patients are serious injuries that can lead to immobility and permanent dependence, negatively impacting patients’ quality of life and resulting in a financial burden for health systems and societies^7–10^. Hip fractures can also lead to death. Mortality rates among the elderly following hip fractures range between 14% to 36% within 1 year of the injury^11–19^. During the first three months after hip fracture, elderly patients have a 5- to 8-fold increased risk of dying^20^. The increased mortality risk persists up to ten years^20^. Because of a predicted increase in life expectancy in western countries over the next decades^21–23^, hip fractures and their consequences will have an even larger impact on health systems and societies in the future.

Factors that influence prognosis of elderly patients after hip fracture are age, gender, comorbidities, anticoagulation therapy, and general physical health status at the time of injury^24^. Furthermore, timing of surgery is thought to play an important role regarding survival. Although international clinical practice guidelines recommend surgical treatment of acute hip fracture within 24 to 48 hours after admission^25–27^, these recommendations are still discussed controversially^28–30^. Some researchers argue that early surgery can lead to an increased risk of perioperative complications, including pneumonia, deep venous thrombosis, bleeding, pulmonary embolism, urinary tract infections, and decubital ulcerations because clinicians do not have enough time to optimise patients’ medical conditions preoperatively^29–31^.

The most recent systematic review on this topic was published in 2010^32^. Since then, many well-conducted studies have been published. To provide a comprehensive overview, it is necessary to systematically review the currently available evidence on the impact of timing of surgery in elderly patients with acute hip fracture. In contrast to former reviews that focused exclusively on mortality, we additionally aimed to assess other patient-relevant outcomes, such as perioperative complications, functional capacity, and quality of life. We also explored whether timing of surgery has different effects in different subgroups, e.g., in patients on anticoagulation treatment or patients with poor physical status.

Our systematic review aimed to answer the following questions:

1. In patients aged 60 years or older with an acute hip fracture, what is the impact of timing of surgery on beneficial and harmful outcomes such as mortality, functional capacity, quality of life, and perioperative complications?
2. Do beneficial or harmful treatment effects of timing of surgery vary by subgroups based on patient characteristics (age, sex), physical status (e.g., ASA Physical Status System), and common medical treatments (e.g., anticoagulation treatment)?

## Methods

To answer our research questions, we conducted a systematic review that has been registered with the International Prospective Register of Systematic Reviews (PROSPERO), registration number: CRD42017058216^33^. The study protocol has been published previously^34^. We will summarise the most important methodological steps in the sections below.

### Search Strategy and Criteria

An experienced information specialist searched MEDLINE (Ovid), PubMed (non-MEDLINE content), Embase.com, the Cochrane Library (Wiley), for the period of January 1997 to May 2017, using keywords and medical subject headings for hip fracture surgery, adult patients, and timing factors. To ensure finding all relevant studies on this topic a broad range of synonyms where used for the search (see Appendix 1 for the search strategy). In addition, we searched the World Health Organization (WHO) International Clinical Trials Registry Platform (ICTRP) and ClinicalTrials.gov, as well as reference lists of relevant publications, websites and conference proceedings of orthopaedic and traumatological societies (see Appendix 2).

### Inclusion and Exclusion

Inclusion and exclusion criteria were predetermined in the published protocol^35^. Eligible study designs were randomised controlled trials (RCTs), non-randomised controlled trials, and prospective controlled cohort studies. The populations of interest were adults aged 60 years or older undergoing surgery for acute intra- and extracapsular hip fracture. We also included studies where only a small proportion (<5%) of patients were younger than 60 years. Studies were included only if they compared early and delayed surgery for hip fractures. The primary outcome was all-cause mortality. Secondary outcomes of interest were perioperative complications, functional capacity, and quality of life. Detailed eligibility criteria are presented in Table 1.

**Table 1:** Eligibility criteria for included studies.

Study characteristic	Inclusion	Exclusion
Population	• Studies including at least 95% adults aged 60 years or older who underwent surgery for acute hip fracture (intra- or extracapsular)	• Studies including 5% or more patients younger than 60 years • Studies on patients undergoing surgery for other reasons than hip fracture • Studies on patients with hip fracture not related to acute trauma, with pathological fractures, or with periprosthetic fractures
Intervention	• Early surgery for hip fracture as defined by authors in the primary study	• Studies that do not compare timing of surgery
Control intervention	• Delayed surgery for hip fracture as defined by authors in the primary study	
Outcomes	• All-cause mortality • Severe perioperative complications ○ Pulmonary embolism ○ Pneumonia ○ Deep vein thrombosis ○ Others Other perioperative complications: ○ Urinary tract infection ○ Pressure ulcer ○ Others • Functional capacity • Quality of life	• Studies that do not include at least one of the outcomes listed under the inclusion criteria
Publication language	• English • German	• All other languages
Geography	No limitation	No limitation
Study design	• Randomised controlled trials• Non-randomised trials• Prospective controlled cohort studies	• Case series • case reports • retrospective controlled cohort studies • case-control • studies studies without a control group
Publication type	Any publication reporting primary data	Publications not reporting primary data, or only available as abstracts
Publication date	Studies published from 1997 onwards	Studies published before 1997

### Assessment of Study Quality and Certainty of Evidence

We used the Newcastle-Ottawa-Scale (NOS) to judge the risk of bias in included cohort studies^36^. Two authors independently assessed the risk of selection bias, comparability of groups, adequacy of outcome measurement, and reporting. We resolved disagreements by consensus or involvement of a third review author.

In addition, we assessed the certainty of evidence (CoE) across studies for important outcomes following recommendations of the Grading of Recommendations, Assessment, Development and Evaluation (GRADE) working group^37^. Experts in the field of orthopaedics and traumatology ranked outcomes regarding clinical and patients’ relevance in a modified two-staged Delphi process. They agreed on mortality, quality of life, perioperative complications, and function/mobility as the most important outcomes. For these outcomes, we graded the certainty of evidence and classified it as “high,” “moderate,” “low,” or “very low.” High certainty means we are very confident that the true effect is close to the effect estimate. On the contrary, if the certainty is very low, we assume that the true effect is likely to be significantly different from the effect estimate^37^.

### Data Collection and Abstraction

Two review authors independently reviewed abstracts and full-text articles in two consecutive steps. Disagreements were resolved by consensus or discussion with a third author. Two team members independently extracted relevant information on study design, methods, patient characteristics, intervention, control, and outcomes from included studies. In case information about relevant outcomes or study characteristics was missing or unclear, we contacted study authors.

### Meta-analysis Methodology

We used the generic inverse variance method to combine effects of individual observational studies that were adjusted for potential confounders and were rated as low or moderate risk of bias in meta-analyses. We pooled data only if at least three studies used comparable cut-offs for “early” and “delayed” surgery and reported the same outcome. In case the studies reported hazard ratios (HR) or odds ratios (OR), we converted them into risk ratio (RR) using the following formulas for HR: RR = 1 − e ^(HR* ln (1 − P^_0_^))^/P_0_^32^, and for OR: RR = OR/((1 − P_0_) + (P_0_ * OR))^38^; P_0_ means the event rate in the control group. For one study^39^, we were not able to calculate P_0_ because no crude numbers of events were reported, so we used the mean P_0_ from the other included studies to convert OR into RR. We added observational studies with unadjusted results, irrespective of their risk of bias judgment to meta-analyses for sensitivity analyses.

To assess statistical heterogeneity in effects between studies, we calculated the chi-squared statistic and the I^2^ statistic (the proportion of variation in study estimates attributable to heterogeneity rather than due to chance)^40,41^. Due to the limited number of studies included in meta-analyses, no funnel plots could be used to assess publication bias. We used RevMan Version 5.3^42^ for all statistical analyses.

For outcomes for which no meta-analyses were possible, we summarise data narratively. If several studies reported the same outcome but meta-analyses were not possible because of high clinical heterogeneity or because the study was rated high risk of bias, we graphically display results in forest plots without pooled summary estimates.

Because data were not sufficient to conduct subgroup analyses, we summarise these results narratively.

## Results

### Study characteristics

**Figure 1:**
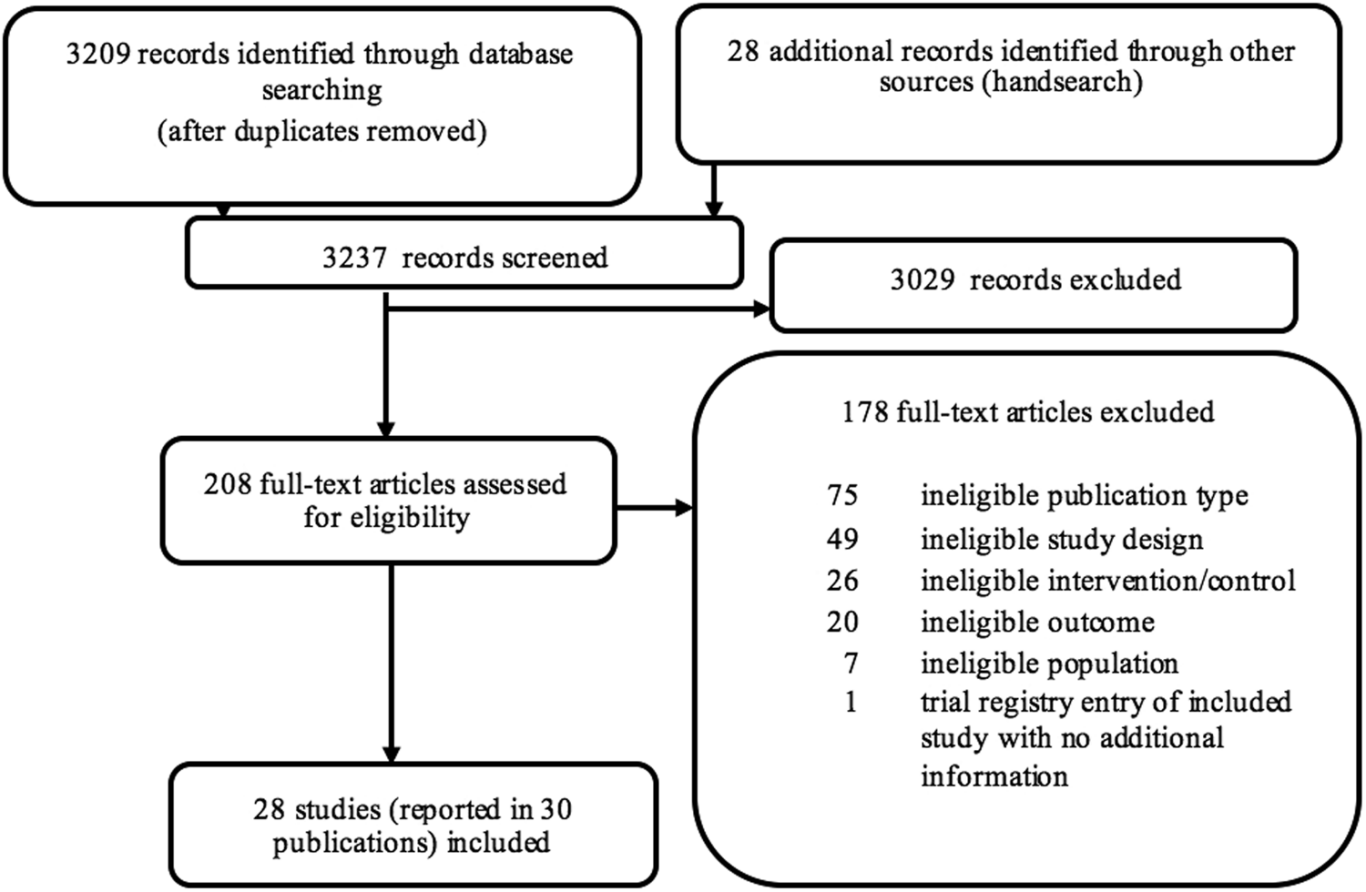
PRISMA flow chart.

We included 28 prospective cohort studies^13,29,31,39,43–67^ (published in 30 articles) reporting results on 31,242 patients (see Fig. 1, PRISMA (Preferred Reporting Items for Systematic Reviews and Meta-Analyses) flowchart). We could not detect any eligible RCTs.

Of the 28 included studies, 15 had a low^29,46,53,57,62,68^ or moderate^13,31,39,45,47,52,55,64–66^ risk of bias, and 13 studies were rated high risk of bias^43,44,48–51,54,56,58–61,63^. Most studies used a cut-off time for surgical delay of 48 or 24 hours; other studies used (additional) cut-offs at 6 hours, 12 hours, 18 hours, 36 hours, and 72 hours. Table 2 presents study and patient characteristics of included studies.

**Table 2:** Characteristics of included studies.

Author, year of publication, country	Follow-up	Number of patients analysed	Age, mean (SD or range)	Female	Fracture type	Comparison early/delayed surgery	Outcomes	Additional information from authors used	Risk of bias
Al-Ani, 2008, Sweden^62^	4 months	744	81 (9)	73%	cervical 49%, trochanteric 43%, subtrochanteric 8%	≤24 h vs. >24 h, ≤36 h vs. >36 h, ≤48 h vs. >48 h	- mortality within 4 months (*adjusted for age*, *sex*, *prefracture walking ability*,*dementia*, *ASA score*), - pressure ulcer (*adjusted for age*, *prefracture walking ability*, *dementia*, *ASA score*,*duration of surgery*)	yes	low
Bretherton, 2015, United Kingdom^53^	12 months	6638	82 (8)	78%	intracapsular 58%, extracapsular 42%	≤6 h vs. >6 h, ≤12 h vs. >12 h, ≤18 h vs. >18 h, ≤24 h vs. >24 h, ≤36 h vs. >36 h, ≤48 h vs. >48 h	- mortality within 1 month *(adjusted for age*, *gender*, *pre-fracture mobility*, *Mini-Mental Test Score*, *fracture type*, *ASA grade*,*prefracture residence)*	no	low
Butler, 2017, Ireland^63^	6 weeks	51	82 (9)	82%	intracapsular 57%, extracapsular 43%	>12 h & ≤36 h vs. >36 h	- functional capacity (Barthel Index) unadjusted	no	high
Crego–Vita, 2017, Spain^44^	24 months	293 (mortality), 136 (function)	83 (65-105)	61%	intracapsular 100%	≤24 h vs. >24 h (mortality) ≤24 h vs. >24 h & ≤72 h vs. >72 h (function)	- mortality within 6 months - mortality within 12 months - mortality within 24 months - functional capacity (FAC level, MBI) - u all outcomes: unadjusted	yes	high
Dailiana, 2013, Greece^39^	12 months	218	79 (7)	64%	intertrochanteric 64%, subcapital 30%, subtrochanteric 6%,	≤48 h vs. >48 h	- mortality within 1 month *(adjusted for age*,*sex*, *Charlson index)* - mortality within 12 months *(adjusted for age*, *sex*, *Charlson index)*	no	moderate
Dorotka, 2003, Austria^48^	6 months	181 (mortality, complications), 152 (function)	early group: 77 (12) delayed group: 79 (12)	76%	Garden type I, II 10%, Garden type III, IV 30%, basocervial 3%, pertrochanteric stable 30%, pertrochanteric unstable 10%, per- and subtrochanteric 17%	≤6 h vs. >6 h, ≤12 h vs. >12 h, >18 h vs. >18 h, ≤24 h vs. >24 h,≤36 h vs. >36 h	- mortality within 6 months perioperative complications (pneumonia) - functional capacity (mobility) - all outcomes: unadjusted	no	high
Elliott, 2003, United Kingdom^49^	12 months	1780	<65 y:12%, 65-75 y: 17%, 75–84 y: 40%, over 85 y: 31%	77%	NR	≤24 h vs. >24 h	- mortality within 12 months (unadjusted)	no	high
Hapuarachchi, 2014, United Kingdom^54^	12 months	146	93 (NR)	84%	femoral neck fractures 100%	≤24 h vs. >24 h, ≤48 h vs. >48 h	- Mortality within 1 months - Perioperative complications all outcomes: unadjusted	no	high
Kelly-Pettersson, 2017, Sweden^64^	12 months	561	82 (10)	72%	femoral neck 54%, intertrochanteric 38%, subtrochanteric 8%	≤24 h vs. >24 h	- Mortality within 12 months - Perioperative complications (pressure ulcer, pneumonia, pulmonary embolus, urinary tract infection) all outcomes: unadjusted	yes (author provided data removing 16 patients younger than 60 years)	moderate
Kim, 2012, South Korea^56^	24 months	415	75 (60–96)	68%	femoral neck 56%, intertrochanteric 44%	≤48 h vs. >48 h	- functional capacity	no	high
Lizaur-Utrilla, 2016, Spain^13^	12 months	628	84 (7)	74%	trochanteric 63%, cervical 37%	≤48 h vs. >48 h	- mortality within 12 months *(adjusted for age*, *gender*, *ASA*, *Charlson index*,*anticoagulation therapy*, *fracture type*,*prosthetic implant*, *complication*,*readmission*, *dementia*, *ADL*, *mobility*, *pre-nursing residence*, *nursing discharge)*	yes	moderate
Maggi, 2010, Italy^65^	6 months	2428	82 (9)	79%	femur neck/head: 56%, intertrochanteric: 37%, subtrochanteric 7%	≤48 h vs. >48 h	- mortality within 6 months (unadjusted, based only on complete follow-up, n = 2,010)	no	moderate
Mariconda, 2015, Italy^52^	12 months	552 (mortality), 568 (complication)	78 (50–105)	77%	femoral neck 42%, trochanteric 55%, subtrochanteric 3%	<72 h vs. ≥72 h	- mortality within 1 month (unadjusted) mortality within 12 months (unadjusted) - perioperative complications within 4 months (*adjusted for Mini-Mental State*,*ASA grade*)	yes	moderate
Moran, 2005, United Kingdom^47^	12 months	2537 (mortality), 2354 (complications)	80 (17–103)	76%	femoral neck 100%	≤24 h vs. >24 h	- mortality within 1 months - perioperative complications (embolism) all outcomes: unadjusted	no	moderate
Muhm, 2013, Germany^51^	12 months	257	84 (NR)	86%	femoral neck 38%, trochanteric 62%	≤48 h vs. >48h–168h	- mortality within 12 months (unadjusted)	yes	high
Orosz, 2004, United States^29^	6 months	1178	82	80.6%	femoral neck 48%	≤24 h vs. >24 h	- mortality within 6 months *(adjusted for age*,*sex*, *nursing home residence*, *independence*,*function*, *comorbidities*, *fracture type*,*hospitalization within* 6 *months*,*hospital site*, *day and time of admission*,*abnormal clinical findings)* - perioperative complications - functional capacity (FIM) *(propensity score matched)*	no	low
Öztürk, 2010, Turkey^59^	12 months	74	78 (8)	70%	NR	≤48 h vs. >48 h	- mortality within 12 months (unadjusted)	no	high
Pajulammi, 2016, Finland^66,67,69^	12 months	1400 (mortality), 611 (function)	84 (65–105)	75%	neck of femur 62%, intertrochanteric 32%, subtrochanteric 6%	≤24 h vs. >24 h	- mortality within 12 months - functional capacity (mobility) all outcomes: unadjusted	yes	moderate
Pioli, 2012, Italy^57,68^	12 months	806	86 (6)	76%	intracapsular 47%, trochanteric 46%, subtrochanteric 7%	≤48 h vs. >48 h	- mortality within 12 months (*adjusted for age*, *sex*, *ADL*, *Charlson index*) - functional capacity (mobility, ADL; unadjusted)	yes	low
Poh, 2013, Singapore^55^	in-hospital (mean 15 days)	242	78 (10)	70%	femoral neck 53%, pertrochanteric 47%	≤48 h vs. >48 h	- perioperative complications (unadjusted)	no	moderate
Rae, 2007, Australia^45^	18 months	222	79 (51–95)	72%	femoral neck 100%	≤24 h vs. >24h–≤48 h	- mortality within 1 months *(adjusted for preoperative length of stay*, *ASA score*,*procedure*, *age*, *theatre cancellations*, *sex)*	no	moderate
Siegmeth, 2005, United Kingdom^46^	12 months	3628	81 (8)	81%	intracapsular 59%, extracapsular 41%	≤48 h vs. >48 h	- mortality within 12 months (unadjusted)	no	low
Smektala, 2000, Germany^50^	12 months	161	84 (NR)	93%	femoral neck NR%, intertrochanteric NR%	≤24 h vs. >24 h	- mortality during hospital stay (unadjusted)	no	high
Smektala, 2008, Germany^31^	12 months	1993 (mortality) 2916 (complications)	82 (7)	80%	femoral neck 50%, pertrochanteric femoral 50%	≤12 h vs. >12h–≤36 h (mortality)≤36 h vs. >36 h (complications)	- mortality within 12 months *(adjusted for age*, *sex*, *time from fracture to surgery*,*ASA*, *MBI*, *comorbidities*, *post-operative complications)* - perioperative complications (pneumonia, embolism, UTI, pressure ulcer; unadjusted)	no	moderate
Trpeski, 2013, Macedonia^43^	6 months	120	74 (10)	78%	inter- and pertrochanteric NR%, subtrochanteric NR%	≤48 h vs. >48 h	- mortality within 1 months - mortality within 6 months all outcomes: unadjusted	no	high
Vertelis, 2009, Lithuania^60^	12 months	265	women 77 (9), men 72 (14)	68%	femoral neck fracture Garden ¾ 100%	≤7 h vs. >7 h	- mortality within 12 months (*adjusted for sex*, *age*, *osteosynthesis*, *arrival to hospital*)	no	high
Vidán, 2011, Spain^58^	in-hospital (median 10 days)	1240 (mortality) 2249 (complications)	84 (7)	82%	femoral neck 41%, intertrochanteric 48%; subtrochanteric 6%; 5% other	≤48 h vs. >48 h (mortality)≤48 h vs. >48 h (complications)	- mortality during hospital stay (*adjusted for age*, *dementia*, *comorbidities*, *ADL*) - perioperative complications (unadjusted)	yes	high
Yonezawa, 2009, Japan^61^	in-hospital (average 39.1 days)	536 (mortality), 347 (function)	83 (9)	83%	trochanteric femoral 52%, femoral neck 48%	≤24 h vs. >24 h	- mortality during hospital stay - functional capacity (mobility) all outcomes: unadjusted	no	high

Abbreviations: ADL, activities of daily living; ASA, American Society of Anaesthesiologists; FAC, Functional Ambulation Categories; FIM, Functional Independence Measure; h, hour; MBI: Modified Barthel Index; NR, not reported; UTI, urinary tract infection; vs., versus.

### Mortality

Overall, 25 studies reported on all-cause mortality: nine studies (14,863 patients) provided adjusted hazard ratios (HR) or odds ratios (OR) for mortality^13,29,31,39,45,53,57,58,62^, adjusting at least for age, sex, and patient’s health status; 16 studies^43,44,46–52,54,59–61,64–66^ (14,654 patients) reported unadjusted effect estimates on mortality.

#### Cut-off 48 hours

**Figure 2:**
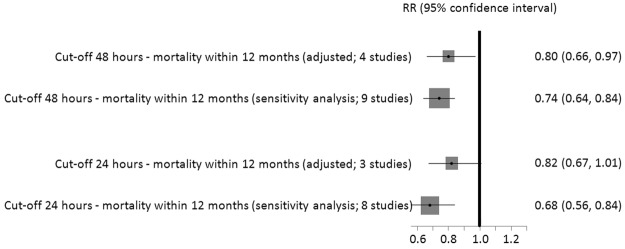
Effects of early and delayed surgery on short- and long-term mortality using 48 hours and 24 hours as cut-offs (summary of results of random-effects meta-analyses and sensitivity analysis).

Based on a meta-analysis of adjusted data from four studies^13,39,62,68^ the absolute risk of dying within 12 months was 21% in patients who had surgery after 48 hours and 17% in patients who had surgery within 48 hours resulting in a 20% smaller long-term mortality risk in patients operated on within 48 hours (RR 0.80, 95% CI 0.66-0.97, 2,396 patients, see Fig. 2). We graded the CoE for this outcome as low. We also conducted sensitivity analyses by adding unadjusted data on mortality from the remaining studies to the meta-analysis, irrespective of their bias risk. Adding the non-adjusted data did not alter the results for long-term mortality (RR 0.74, 95% CI 0.64-0.84, 8,903 patients)^13,39,43,46,51,57,59,62,65,68^ (see Fig. 2). No statistically significant differences were observed in two studies presenting adjusted data on short-term mortality (within 1 months) (RR 0.89, 95% CI 0.59-1.35, 6,638 patients; RR 0.85, 95% CI 0.66-1.10, 218 patients; CoE: very low)^39,53^. In sensitivity analyses, including unadjusted data, surgery within 48 hours was associated with a statistical significant benefit on short-term mortality (RR 0.78, 95% CI 0.62-0.98, 9,371 patients)^39,43,53,54,58^ (see Appendix 3, Fig. 5).

**Figure 3:**
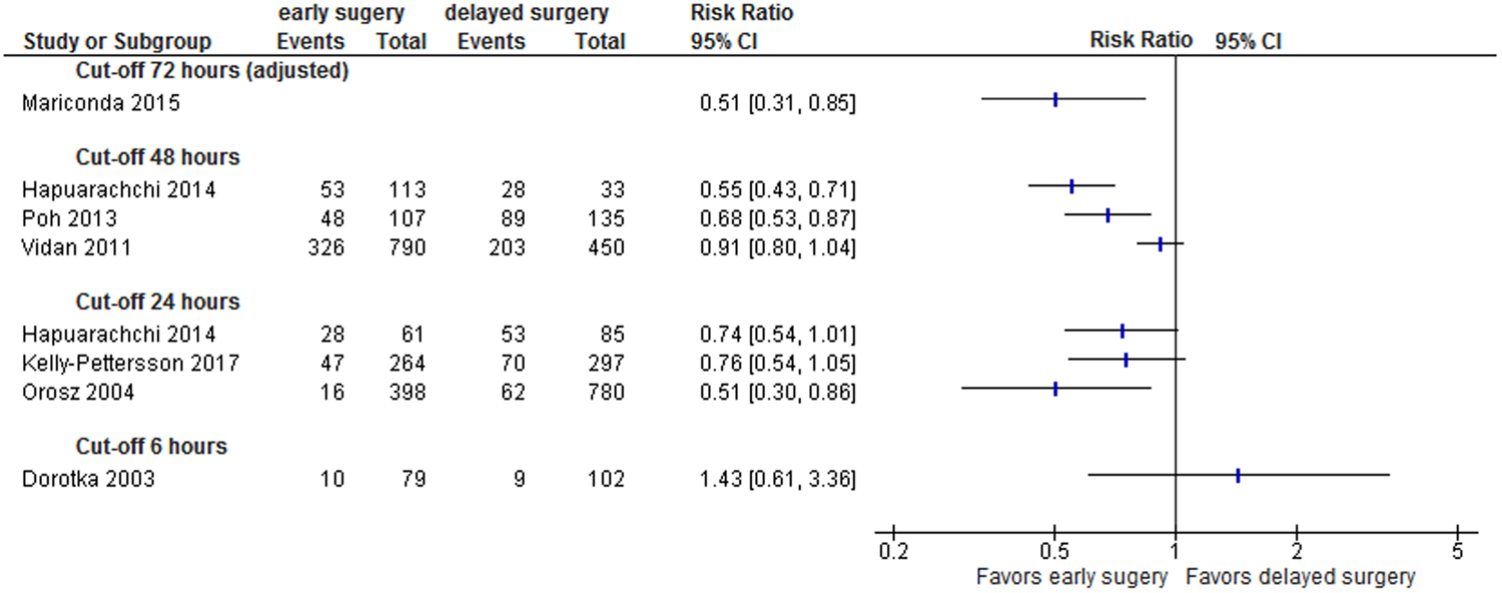
Perioperative complications (adjusted and unadjusted data); Mariconda 2015: effect estimate presented is odds ratio (OR) not RR and based on adjusted data so no event rates displayed; Abbreviations: CI: confidence interval.

**Figure 4:**
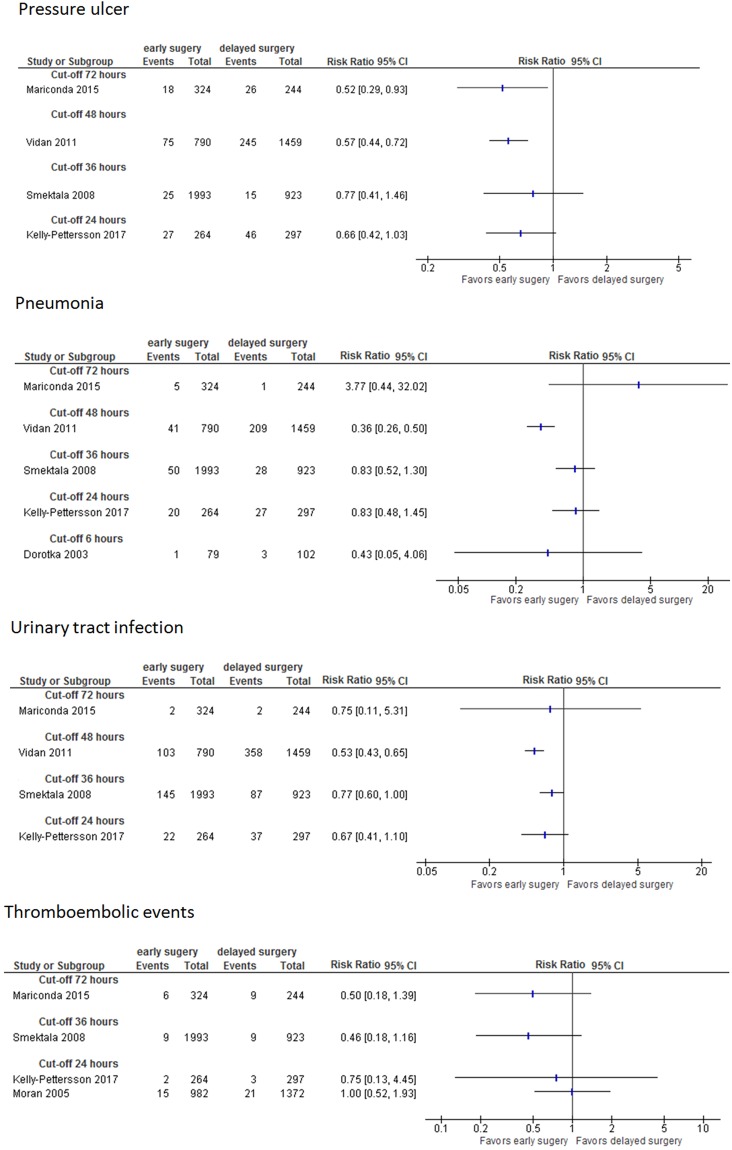
Pneumonia, pressure ulcers, urinary tract infection, thromboembolic events (unadjusted data); Abbreviations: CI: confidence interval.

**Figure 5:**
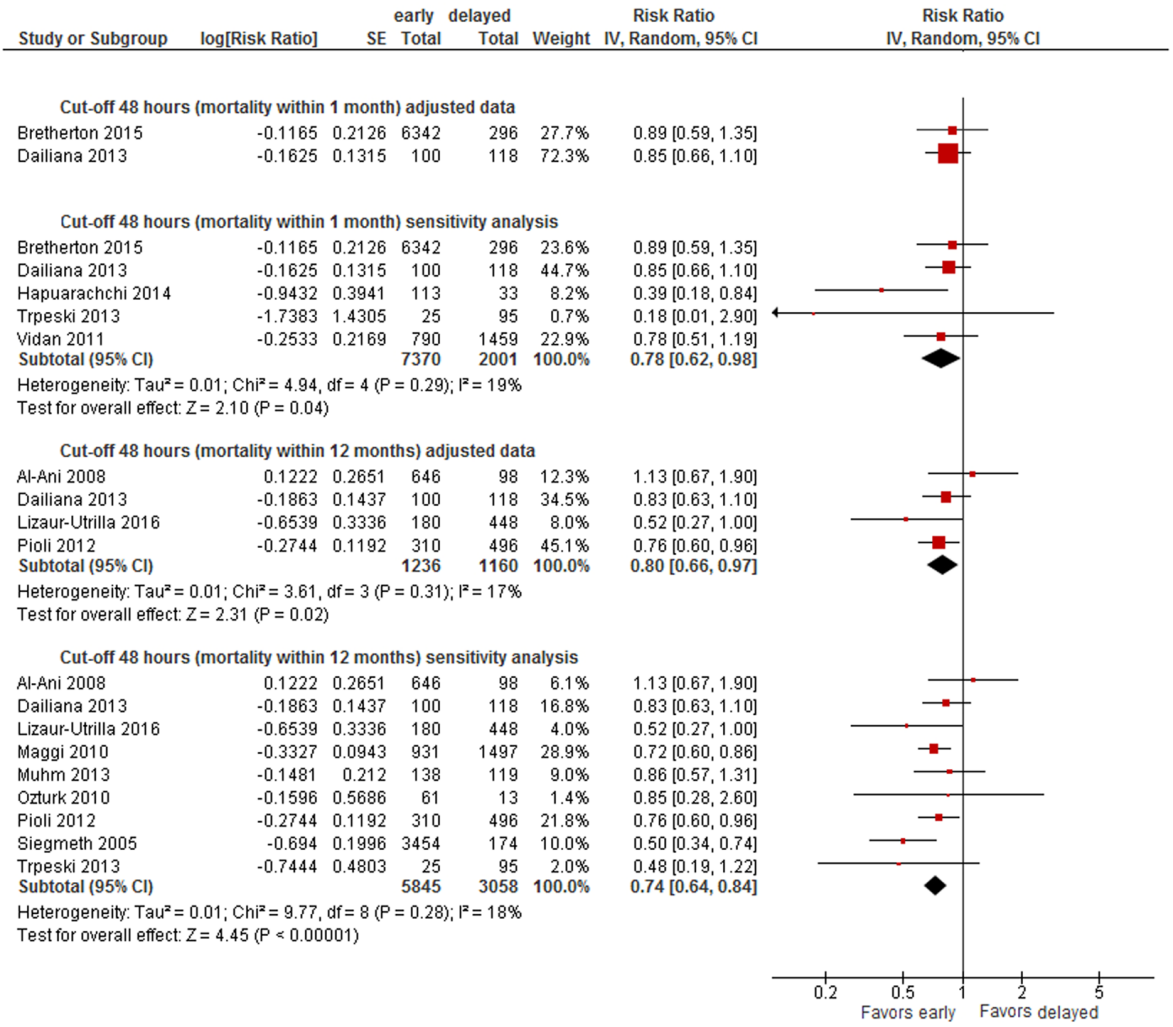
Cut-off 48 hours - short- and long-term mortality adjusted and sensitivity analyses incl. unadjusted data.

#### Cut-off 24 hours

A meta-analysis of three trials^29,62,65^(2,853 patients) rendered an 18% lower risk of long-term mortality in patients operated on within 24 hours (within 12 months: RR 0.82, 95% CI 0.67-1.01, CoE: low, absolute mortality risk early surgery: 17%, delayed: 14%) (see Fig. 2). When adding unadjusted data in sensitivity analyses, the difference between surgery within and after 24 hours was statistically significant (RR 0.68, 95% CI 0.56-0.84, 7,069 patients)^29,44,48,49,62,64,65,69^ (see Fig. 2). No statistically significant differences were observed in two studies presenting adjusted data on short-term mortality (RR 1.03, 95% CI 0.84-1.26, 6,638 patients; RR 0.85, 95% CI 0.29 to 2.49, 222 patients; CoE: very low)^45,53^, as well as in sensitivity analyses (RR 1.04, 95% CI 0.85-1.29)^31,45,47,53,54,61^ (see Appendix 3, Fig. 7).

**Figure 6:**
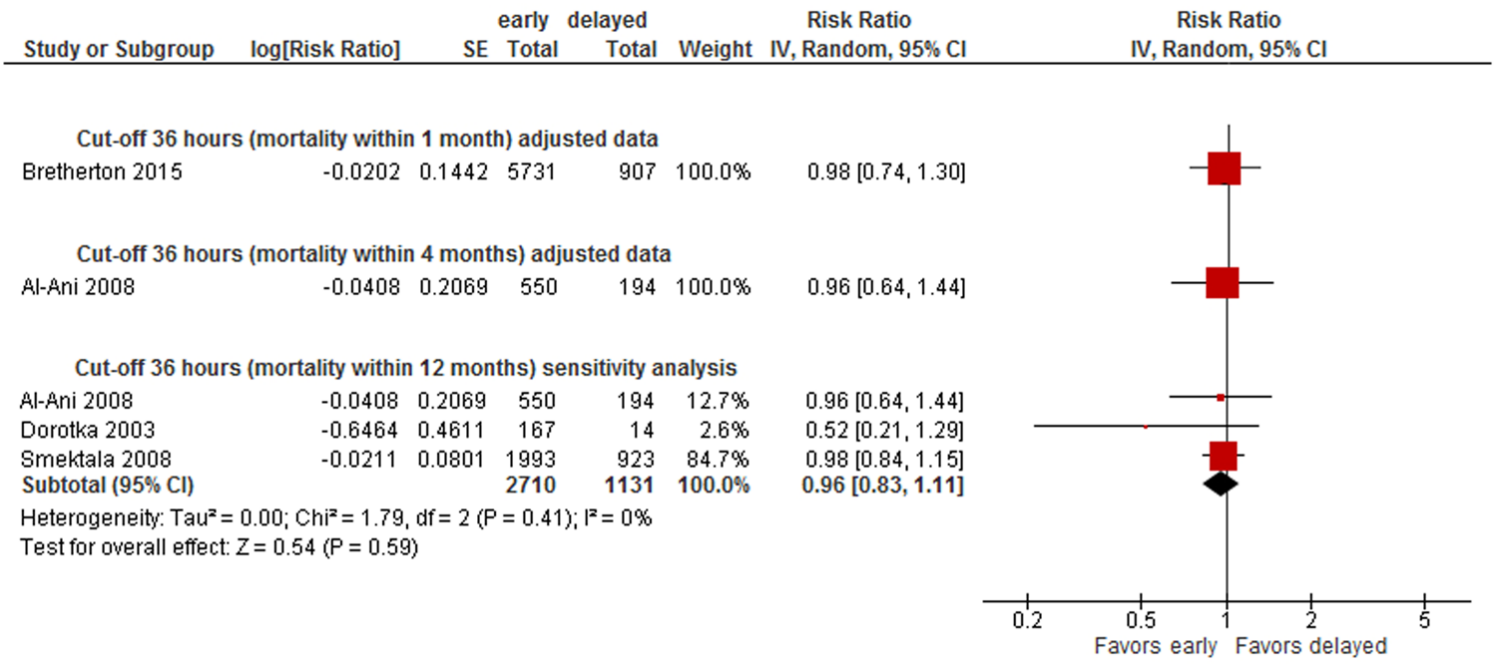
Cut-off 36 hours - short- and long-term mortality adjusted and sensitivity analyses incl. unadjusted data.

**Figure 7:**
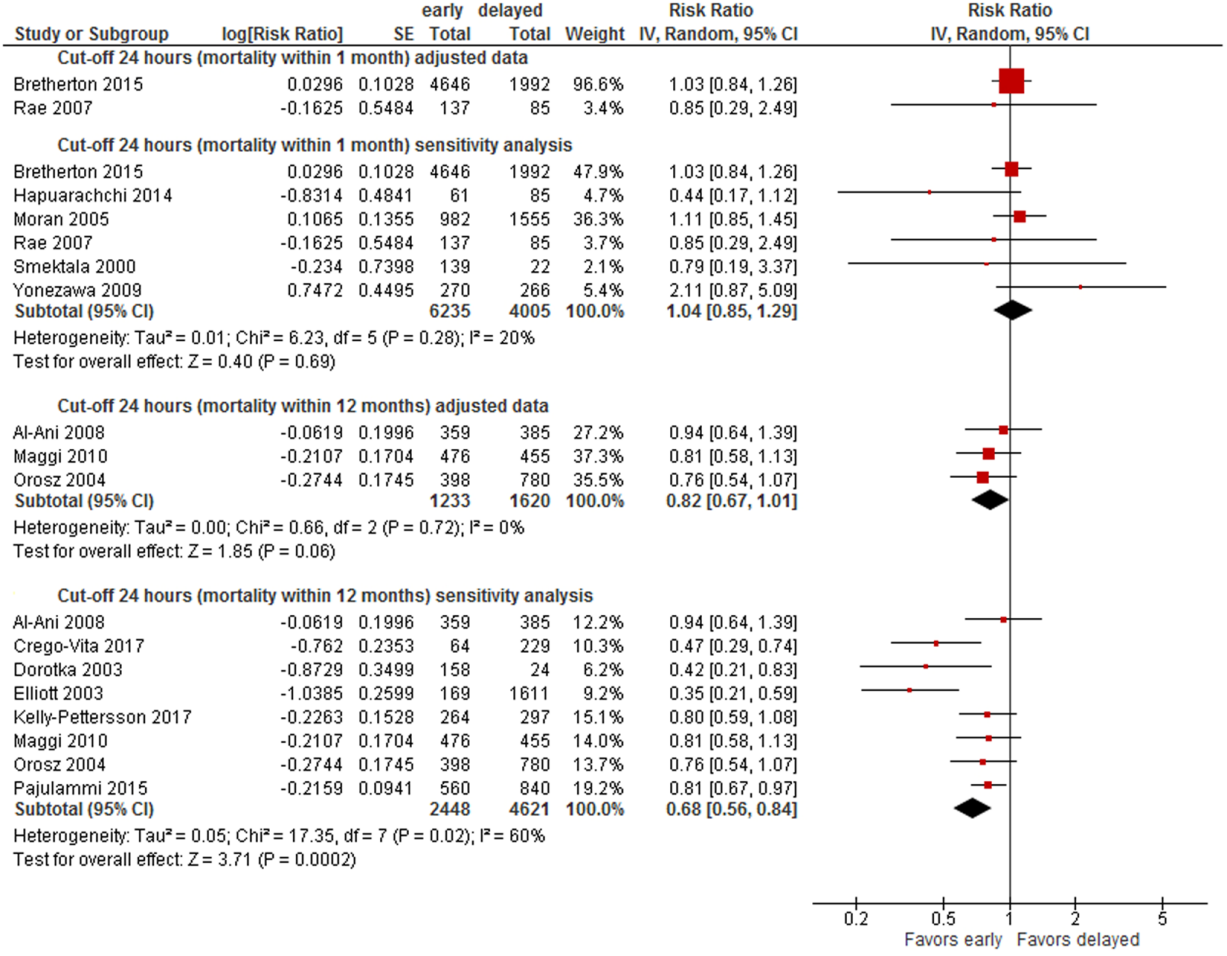
Cut-off 24 hours - short- and long-term mortality adjusted and sensitivity analyses incl. unadjusted data.

Figure 2 summarises results of meta-analyses on long-term mortality and corresponding sensitivity analyses.

**Figure 8:**
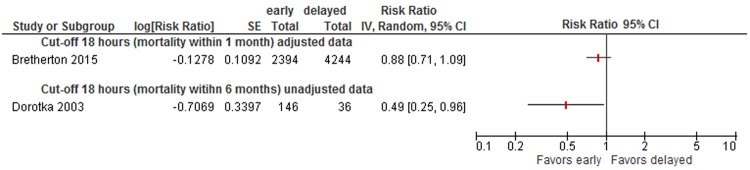
Cut-off 18 hours - short- and long-term mortality adjusted and unadjusted data (not pooled).

**Figure 9:**
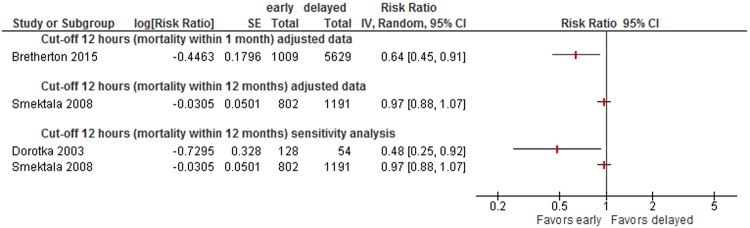
Cut-off 12 hours - short- and long-term mortality adjusted and unadjusted data (not pooled).

**Figure 10:**
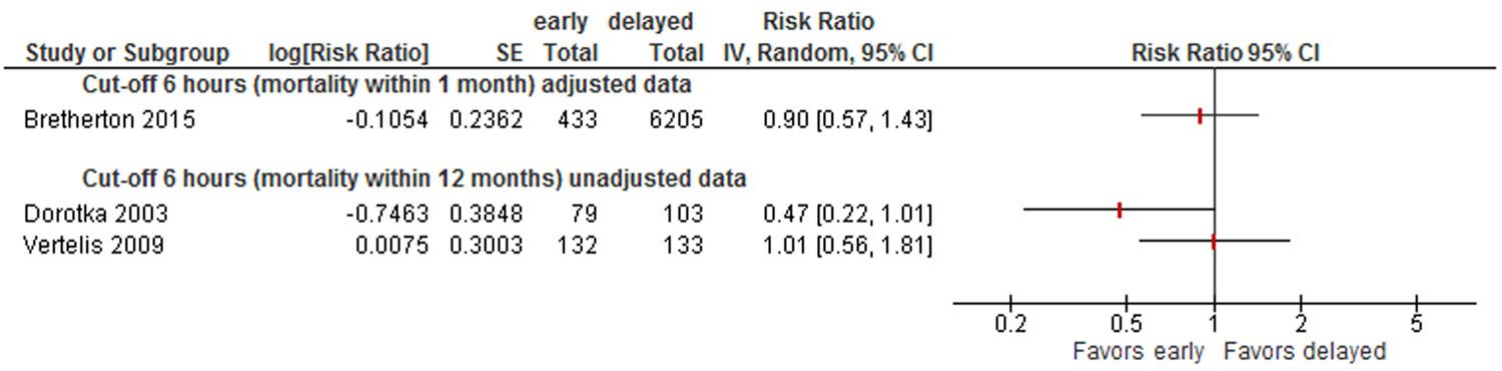
Cut-off 6 hours - short- and long-term mortality adjusted and unadjusted data.

**Figure 11:**
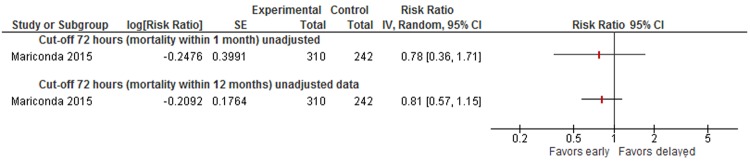
Cut-off 72 hours - short- and long-term mortality adjusted and unadjusted data.

Data were insufficient to conduct meta-analyses for other cut-offs (6, 12, 18, 36, 72 hours) of timing of surgery. However, to illustrate the results on mortality of all studies, we present forest plots for each cut-off in Appendix 3 (48 hours: see Fig. 5, 36 hours: see Fig. 6, 24 hours: see Fig. 7, 18 hours: see Fig. 8, 12 hours: see Figs 9, 6 hours: see Fig. 10, 72 hours: see Fig. 11).

### Perioperative Complications

Two studies with low^62^ and medium^52^ risk of bias reported adjusted data on general perioperative complications or pressure ulcers, respectively. Mariconda *et al*. reported data on 568 patients and showed that surgery within 72 hours was associated with decreased odds of general complications such as pressure ulcers, urinary tract infection, deep vein thrombosis/embolism, or stroke (absolute risk of complications: 17% vs. 8%; OR 0.51, 95% CI 0.31-0.85, see Fig. 3)^52^.

Six studies reported unadjusted data for perioperative complications^29,48,54,55,58,64^. Figure 3 presents unadjusted effect estimates of individual studies. While a cut-off of 6 hours did not show significantly different rates of complications, patients who had surgery within 24 or 48 hours suffered from complications less frequently than those with late surgery.

One study on 744 patients used three different cut-offs (24, 36, 48 hours) for “delayed surgery” and presented adjusted data for pressure ulcers. The odds of developing pressure ulcers increased with the time of delay (>24 hours: OR 2.19, 95% CI 1.21-3.96; >36 hours: OR 3.42, 95% CI 1.94-6.04; >48 hours: OR 4.34, 95% CI 2.34-8.04)^62^. In studies reporting unadjusted data the risk for developing pressure ulcers, pneumonia, urinary tract infections, or thromboembolic events was either smaller for patients who had early surgery or similar between both groups; it was not higher in any study for patients who had early surgery (see Fig. 4). CoE for perioperative complications was very low.

### Quality of life

None of the included studies reported how timing of surgery affects the patients’ quality of life.

### Functional capacity

Measuring of mobility and functional capacity was different among eight studies^29,44,48,56,57,61,63,66^ and data are summarised in Table 3. Patients who had early surgery had similar or slightly better functional capacity compared to those operated on later (CoE: very low).

**Table 3:** Functional capacity outcomes.

Study, year	Function/Mobility outcome	Cut-off	Outcome in patients operated on early		Outcome in patients operated on delayed	
Continuous outcome measure			Mean score (measure of dispersion)	N	Mean score (measure of dispersion)	N
Crego-Vita, 2017^44^	FAC* (functional ambulation category) at 6 months	≤24 h vs. >24 h–≤72 h	4 (NR)	64	3 (NR)	72
Crego-Vita, 2017^44^	FAC* (functional ambulation category) at 12 months	≤24 h vs. >24 h–≤72 h	4 (NR)	64	4 (NR)	72
Crego-Vita, 2017^44^	FAC* (functional ambulation category) at 2 years	≤24 h vs. >24 h–≤72 h	3 (NR)	64	3 (NR)	72
Crego-Vita, 2017^44^	MBI** (Modified Barthel Index) at 6 months	≤24 h vs. >24 h–≤72 h	60 (NR)	64	48 (NR)	72
Crego-Vita, 2017^44^	MBI** (Modified Barthel Index) at 12 months	≤24 h vs. >24 h–≤72 h	71 (NR)	64	58 (NR)	72
Crego-Vita, 2017^44^	MBI** (Modified Barthel Index) at 2 years	≤24 h vs. >24 h–≤72 h	69 (NR)	64	55 (NR)	72
Orosz, 2006^29^	FIM*** (Functional independence measure) locomotion (range 2–14) at 6 months *(propensity score matched*, 296 *pairs)*	24 h	9.4 (NR)	398	9.3 (NR)	780
Orosz, 2006^29^	FIM*** (Functional independence measure) self-care (range 6–42) at 6 months *(propensity score matched*, 299 *pairs)*	24 h	32.3 (NR)	398	33.4 (NR)	780
Orosz, 2006^29^	FIM*** (Functional independence measure) transferring (range 3–21) at 6 months *(propensity score matched*, 302 *pairs)*	24 h	14.4 (NR)	398	14.9 (NR)	780
Butler, 2017^63^	Barthel Index** (mean decrease)	36 h	10 (IQR 0–19)	30	30 (IQR 25–40)	21
Pioli, 2012^57^	ADL**** (activities of daily living) at 6th months	48 h	3.1 (SD ± 2.1)	310	3.4 (SD ± 2.2)	496
**Dichotomous outcome measure**			**Proportion**	**N**	**Proportion**	**N**
Dorotka, 2003^48^	Mobility at 6 months (no walking aids needed)	6 h	33% (NR)	71	23% (NR)	81
Pajulammi, 2015^66^	Same or better mobility level at 1 year	24 h	65% (NR)	258	60% (NR)	353
Yonezawa, 2008^61^	Mobility in those independent before injury	24 h	52% (NR)	173	41% (NR)	174
Pioli, 2012^57^	Independent walking at 6 months	48 h	42% (NR)	310	39% (NR)	496
Kim, 2012^56^	Recovery to former functional capacity (2 years after surgery)	48 h	45% (NR)	174	34% (NR)	241

Abbreviations: ADL, activities of daily living; FAC, Functional Ambulation Categories; FIM, Functional Independence Measure; h, hour; IQR, interquartile range; MBI: Modified Barthel Index; N, total number of patients in this group; NR, not reported; SD, standard deviation.

*FAC scale from 1–5; higher score indicates independence.

**MBI scale from 0–100; higher score indicates independence.

***FIM, range of scale depends on subscale; higher score indicates independence.

****Higher score indicates independence.

### Impact of timing of surgery in subgroups

Due to insufficient data, we were not able to conduct subgroup analyses to assess different effects of timing of surgery between age groups, sex, patients’ physical status, and anticoagulation. However, six studies assessed the effects of timing of surgery in different subgroups^29,47,54,57,58,61^. Below we present results narratively.

#### Age

In two studies, timing of surgery (before or after 24 hours) showed no significant difference in mortality rates in different age groups. Yonezawa *et al*. showed that there was no statistically significant difference in mortality in patients 85 years and older, whether they had surgery within 24 hours or later (early: 10/136; 7% vs. delayed: 5/117; 4%; p = 0.301), as well as in patients younger than 85 years (early: 5/134; 4% vs. delayed: 2/149; 1%; p = 0.363)^61^. Vidán *et al*. also reported that time to surgery and age showed no interaction (p = 0.500)^58^.

#### Sex

In male patients, early surgery (within 24 hours) was associated with higher mortality (6/40; 15% vs. 1/51; 2%; p = 0.040), in females it was not (9/230; 4% vs. 6/215; 3%; p = 0.512)^61^. However, event rates are very small, and the observed differences could be chance findings.

#### Physical status

Timing of surgery (before or after 24 hours) was associated with similar mortality rates in dependently (early: 6/173; 4% vs. delayed: 3/174; 2%; p = 0.494) and independently living patients (early: 9/96; 9% vs. delayed: 4/90; 4%; p = 0.188). Patients with comorbidities benefited more often from surgery within 24 hours (early: 3/196; 7% vs. delayed 5/200; 3%; p = 0.048). In medically fit patients without comorbidities no statistically significant difference between early and delayed was detected^61^. Again, the low number of events makes chance findings inevitable.

Another study divided patients into two groups, either fit or unfit for immediate surgery, depending on their physical status. In the group of patients considered fit for surgery, no statistically significant difference between early (within 24 hours) and delayed surgery was observed regarding 30-day mortality (85/982; 9% vs. 85/1166; 7%; p = 0.510)^47^. In the group of patients with acute medical comorbidities, there was no significant relationship between timing of the surgery and mortality at 30 days, 90 days, or one year (HR 0.68, 95% CI 0.34-1.39; p = 0.290; HR 1.16, 95% CI 0.72-1.86; p = 0.540; HR 1.03, 95% CI 0.68-1.58; p = 0.880, respectively). A delay of more than one day from injury to presentation was associated with higher mortality in this group of patients (HR 2.1, 95% CI 1.01-4.2; p = 0.048)^47^.

Hapuarachchi *et al*. included 146 patients at the age of 90 or older^54^ and stratified patients according to the orthopaedic POSSUM (The Physiological and Operative Severity Score for enUmeration of Mortality and morbidity) score. Mortality was statistically significant higher in patients with POSSUM scores of ≥42 and delayed surgery (after 48 hours) as compared with early surgery (within 48 hours): early: 7% vs. delayed: 50%; p = 0.009. In patients with lower POSSUM scores no difference in mortality between early (within 48 hours) and delayed surgery was reported (POSSUM score 37-40: early: 8% vs. delayed: 11%, p = 0.500; POSSUM score ≤ 36: early: 24% vs. delayed: 50%, p = 0.310).

Pioli *et al*. hypothesised that timing of surgery is more important for frail elderly patients than for older people without functional impairment. Therefore, they divided patients into three groups according to their IADL (Instrumental Activities of Daily Living) score. One-year mortality in group 1 (dependent) and group 2 (intermediate level) relatively increased by 14% and 21%, respectively, per day of surgical delay (HR 1.14; 95% CI 1.06-1.22, p < 0.001 and HR 1.21; 95% CI 1.09-1.34, p < 0.001), but not in group 3 (high independence; HR 1.05; 95% CI 0.79-1.41, p = 0.706)^57^.

In a prospective cohort study including 1,206 patients, those with abnormal clinical findings or the need for further preoperative evaluation were excluded to form a restricted cohort of medically fit patients. In this group, early surgery within 24 hours had no association with functional outcomes or mortality, but was associated with reduced major postoperative complications (p = 0.041)^29^.

#### Anticoagulation treatment

In most of the studies, anticoagulants were more common in the delayed group and frequently caused surgical delay^13,39,48,51^. However, we did not identify any study reporting on differences between early and delayed surgery in patients with and without anticoagulation treatment.

## Discussion

To the best of our knowledge, this is the first systematic review critically assessing all relevant prospective studies on this topic since 2010. We identified 20 new studies that had not been considered in the previous reviews^32,70,71^. Our findings agree with previous systematic reviews. Simunovic *et al*. showed that early surgery (within 24 to 72 hours) can reduce the risk of all-cause mortality in patients aged 60 or older by 19% (risk ratio (RR) 0.81, 95% confidence interval (CI) 0.68–0.96)^32^. Early surgery was also associated with a reduction of pressure ulcers and postoperative pneumonia (RR 0.48, 95% CI 0.34-0.69)^32^. Another systematic review including prospective and retrospective observational studies also demonstrated that a delay in surgery beyond 48 hours was associated with an increased 1-year-mortality and 30-day mortality risk (odds ratio (OR) 1.32, 95% CI 1.21-1.43; 30-day mortality: OR 1.41, 95% CI 1.29–1.54)^70^.

In contrast to other systematic reviews we looked at the effect of different cut-offs for “early” and “delayed” surgery separately and found that early surgery within 48 hours was associated with decreased long-term mortality in elderly patients after hip fractures. Single studies using other cut-offs (6, 12, 18, 24 or 36 hours) did not demonstrate significant differences in mortality between early and delayed surgery. However, these studies were probably underpowered and it is important to note that no study demonstrated a beneficial effect of delayed surgery on mortality.

Although findings of this review strengthen existing guidelines recommending surgery within 48 hours, in clinical practice, delay of surgery of hip fractures is quite common. In situations where patients need medical optimisation due to poor health status or long-term medication^72^, delays cannot be avoided. However, the reasons for delayed surgery are also often limited capacity of operating rooms and personnel, or weekend and holiday administration^32,58,73,74^. Cha *et al*. showed that hospital factors are accountable for three-fourths of the surgical delays^74^. In the interest of high quality care, organisational and structural improvements, such as better availability of operating rooms and staff, are necessary to enable early surgery. There is also general agreement that rapidly correctable comorbidities such as anaemia, hypovolemia, electrolyte imbalance, and correctable cardiac arrhythmias should not delay the operation^27^.

Only six of the included studies reported the effects of time to surgery in our predefined subgroups. In healthy, independent patients, delayed surgery was not as problematic as in patients with comorbidities. In most of these studies, the event rate was very small. Hence, the results could be chance findings. Moreover, the studies presented only unadjusted data. It should be emphasised that conclusions based on this data must be drawn carefully. Nevertheless, if availability of staff and operation room is limited, comorbid patients could be prioritised and have early surgery, presupposing that they do not have clear contraindications for surgery.

Our study has some limitations. We graded the certainty of evidence for all outcomes low or very low, which means that our confidence in the findings is limited. One reason for the low certainty of evidence is that we only identified prospective cohort studies but no RCTs. Results of observational studies must be interpreted with caution since confounding could distort the findings. It is possible that non-organisational reasons for delay of surgery such as need for medical optimisation also increased the risk of dying, independently, or in addition to timing of surgery. To minimise the distortion through confounding we included for our main analysis only data from adjusted analyses where at least the most important confounders such as age, gender, ASA score, fracture type and comorbidities had been considered. However, due to lack of randomisation, confounding cannot be completely eliminated.

The studies identified used different cut-offs to define early and delayed surgery. We combined only data from studies using very similar cut-offs. This allowed us to include only a small number of the included studies into meta-analyses. However, presenting the evidence for different cut-offs separately is relevant to inform clinical practice about the optimal timing of surgery.

No study conducted subgroup analysis with tests for interaction. However, some analysed the effect of timing of surgery in separate strata, allowing us to draw some conclusions about different effects in subgroups. Moreover, often the number of events was very small, making chance findings very likely. The results on subgroups therefore have to be interpreted with caution.

Despite our comprehensive search, it is possible that not all studies conducted on this topic have been detected (e.g., studies published in languages other than English or German). Publication bias cannot be ruled out, and we were not able to assess potential publication bias with a funnel plot. However, we contacted experts in the field, searched trial registries, and ultimately found 20 new studies that have not been included in former systematic reviews.

To overcome the limitation of observational studies, RCTs on this topic are needed. Although experts often argue, that this is unethical and not possible to implement, a RCT on timing of surgery in hip fracture patients is on the way. The HIP-ATTACK trial (HIP fracture Accelerated surgical TreaTment And Care tracK) will compare the effect of accelerated surgery and standard surgical care on perioperative complications and mortality^75^. A total of 1,200 patients older than 45 with low-energy hip fracture will be included in the study. The results of this trial will inform clinical practice and for the first time control adequately for known and unknown confounders.

## Conclusion

In elderly patients sustaining hip fracture, early surgery is associated with reduced mortality and perioperative complications. Patients operated on within 48 hours had a 20% lower 1-year mortality.

However, timing of surgery for patients with hip fractures remains a challenge, as it requires multidisciplinary coordination between different occupational groups and the availability of appropriate surgical capacity with competent staff and proper equipment. No study demonstrated a survival benefit with delayed surgery. Future studies should investigate the effect of early surgery in subgroups of patients (e.g. patients with greater co-morbidities or anticoagulation treatment) and include data on patient-relevant outcomes, such as quality of life measurements. Furthermore, randomised controlled trials are needed to rule out potential confounding.

## Electronic supplementary material

Appendices

## Data Availability

The datasets generated during the study are available from the corresponding author on reasonable request.
