# Supplementary material for: Impact of timing of surgery in elderly hip fracture patients: a systematic review and meta-analysis
Source: Sci Rep. 2018 Sep 17;8:13933. doi: 10.1038/s41598-018-32098-7 (PMC6141544; doi:10.1038/s41598-018-32098-7)
Supplement: Supplementary file 1 — Appendices [file 41598_2018_32098_MOESM1_ESM.docx]

**Impact of timing of surgery in elderly hip fracture patients: a systematic review and meta-analysis**

**Running title: Time-to-surgery in hip fracture patients**

Study Design: Systematic Review and Meta-Analysis

Thomas Klestil^1,2,*^, Christoph Röder^2^, Christoph Stotter^2,3^, Birgit Winkler^2^, Stefan Nehrer^3, 4^, Martin Lutz^5^, Irma Klerings^6^, Gernot Wagner^6^, Gerald Gartlehner^7,8^, Barbara Nussbaumer-Streit^6,7^

^1^ Danube University Krems, Faculty of Health and Medicine, Department for Health Sciences and Biomedicine, Center for Medical Specialisations, Dr. Karl-Dorrek-Str. 30, A-3500 Krems

^2^ LK Baden-Mödling-Hainburg, Department of Orthopedics and Traumatology, Waltersdorferstraße 75, A-2500 Baden, Austria

^3^ Danube University Krems, Faculty of Health and Medicine, Department for Health Sciences and Biomedicine, Center for Regenerative Medicine and Orthopedics, Dr. Karl-Dorrek-Str. 30, A-3500 Krems

^4^ UK Krems, Department of Orthopedic Surgery, Mitterweg 10, A-3500 Krems, Austria

^5^ Landeskrankenhaus Hall, Department of Orthopedics and Traumatology, Milser Straße 10, A-6060, Hall in Tirol, Austria

^6^ Danube University Krems, Department of Evidence-based Medicine and Clinical Epidemiology, Dr. Karl-Dorrek-Str. 30, A-3500 Krems, Austria

^7^ Cochrane Austria, Danube University Krems, Dr. Karl-Dorrek-Str. 30, A-3500 Krems, Austria

^8^ RTI International, 3040 Cornwallis Road, Research Triangle Park, NC, 27790, United States

^*^ [thomas.klestil@donau-uni.ac.at](mailto:thomas.klestil@donau-uni.ac.at)

Investigation performed at Danube-University Krems, Austria

**Appendix 1**

**Database searches**

Ovid MEDLINE(R) 1946 to April Week 4 2017, Ovid MEDLINE(R) Epub Ahead of Print May 03, 2017, Ovid MEDLINE(R) In-Process & Other Non-Indexed Citations May 03, 2017, Ovid MEDLINE(R) Daily Update May 03, 2017

04 May 2017

| **#** | **Suchen** | **Ergebnisse** |
| --- | --- | --- |
| 1 | exp Femoral Fractures/co, mo, su [Complications, Mortality, Surgery] | 20166 |
| 2 | ((hip or femoral or femur or pertrochanteric or subtrochanteric or intertrochanteric or intracapsular or extracapsular) adj1 fractur*).ti,ab. | 22201 |
| 3 | 1 or 2 | 33574 |
| 4 | Time Factors/ | 1113433 |
| 5 | ((delay* or time or timing or early or earlier) adj2 (surgery or surgical* or operat*)).ti,ab. | 95678 |
| 6 | ((hour* or day?) adj4 (surgery or surgical* or operat*)).ti,ab. | 75720 |
| 7 | 4 or 5 or 6 | 1254747 |
| 8 | 3 and 7 | 4388 |
| 9 | exp animals/ not exp humans/ | 4396418 |
| 10 | 8 not 9 | 4207 |
| 11 | exp age groups/ not exp aged/ | 5420379 |
| 12 | 10 not 11 | 3417 |
| 13 | (english or german).lg. | 23952988 |
| 14 | 12 and 13 | 3011 |
| 15 | limit 14 to yr="1997 -Current" | 2424 |
| 16 | remove duplicates from 15 | 2282 |

PubMed

03 May 2017

| Search | Query | Items found |
| --- | --- | --- |
| #1 | Search hip fractur*[tiab] OR femoral fractur*[tiab] OR femur fractur*[tiab] OR pertrochanteric fractur*[tiab] OR subtrochanteric fractur*[tiab] OR intertrochanteric fractur*[tiab] OR intracapsular fractur*[tiab] OR extracapsular fractur*[tiab] | 22708 |
| #2 | Search delay*[ti] OR time[ti] OR timing[ti] OR hour*[ti] OR day[ti] OR days[ti] OR early[ti] OR earlier[ti] | 595747 |
| #3 | Search surgery[ti] OR surgical*[ti] OR operativ*[ti] | 555499 |
| #4 | Search (#2 AND #3) | 19469 |
| #5 | Search delayed surgery[tiab] | 960 |
| #6 | Search early surgery[tiab] | 2763 |
| #7 | Search timing[tiab] AND (surgery[tiab] OR surgical*[tiab]) | 13805 |
| #8 | Search (#4 OR #5 OR #6 OR #7) | 33855 |
| #9 | Search (#1 AND #8) | 443 |
| #10 | Search ("1997"[Date - Publication] : "3000"[Date - Publication]) | 15208262 |
| #11 | Search (#9 AND #10) | 403 |
| #12 | Search "english"[Language] OR "german"[Language] | 23403911 |
| #13 | Search (#11 AND #12) | 387 |
| #14 | Search pubmednotmedline [sb] OR pmcbook OR (publisher [sb] AND (pubstatusnihms OR pubstatuspmcsd)) | 1956492 |
| #15 | Search (#13 AND #14) | 40 |

Embase.com (Elsevier)

04 May 2017

| No. | Query | Results |
| --- | --- | --- |
| #1 | 'femur fracture'/exp/dm_co,dm_dm,dm_si,dm_su,dm_th | 18084 |
| #2 | ((hip OR femoral OR femur OR pertrochanteric OR subtrochanteric OR intertrochanteric OR intracapsular OR extracapsular) NEAR/1 fractur*):ti,ab | 28405 |
| #3 | #1 OR #2 | 40030 |
| #4 | 'time factor'/exp | 8738 |
| #5 | ((delay* OR time OR timing OR early OR earlier) NEAR/2 (surgery OR surgical* OR operat*)):ti,ab | 137988 |
| #6 | ((hour* OR day?) NEAR/4 (surgery OR surgical* OR operat*)):ti,ab | 74588 |
| #7 | #4 OR #5 OR #6 | 211130 |
| #8 | #3 AND #7 | 2890 |
| #9 | 'animal'/exp NOT 'human'/exp | 4825097 |
| #10 | #8 NOT #9 | 2832 |
| #11 | 'groups by age'/exp NOT 'aged'/exp | 6303495 |
| #12 | #10 NOT #11 | 2438 |
| #13 | #12 AND ([english]/lim OR [german]/lim) | 2100 |
| #14 | #13 AND [1997-2017]/py | 1911 |

Cochrane Library (Wiley)

04 May 2017

| ID | Search | Hits |
| --- | --- | --- |
| #1 | [mh "Femoral Fractures"/co,mo,su] | 989 |
| #2 | ((hip or femoral or femur or pertrochanteric or subtrochanteric or intertrochanteric or intracapsular or extracapsular) near/1 fractur*):ti,ab,kw | 3045 |
| #3 | #1 or #2 | 3225 |
| #4 | [mh "Time Factors"] | 59230 |
| #5 | ((delay* or time or timing or hour* or day or days or early or earlier) near/4 (surgery or surgical* or operat*)):ti,ab | 24242 |
| #6 | #4 or #5 | 81143 |
| #7 | #3 and #6 | 506 |
| #8 | #7 Publication Year from 1997 to 2017 | 434 |

**Appendix 2**

**Additional searches**

World Health Organization (WHO) International Clinical Trials Registry Platform (ICTRP)

04 May 2017

| 18 records for 18 trials found for: | hip fracture AND timing OR hip fracture AND delay OR hip fracture AND waiting OR hip fracture AND early |
| --- | --- |

ClinicalTrials.gov

04 May 2017

| 59 studies found for: | timing OR waiting OR delay OR delayed OR early \| (hip OR femoral OR femur OR pertrochanteric OR subtrochanteric OR intertrochanteric OR intracapsular OR extracapsular) AND (fracture OR fractures) |
| --- | --- |

Websites and conference proceedings of orthopedic and traumatological societies (all 2008 to 2017)

- Orthopaedic Trauma Association
- International Society of Orthopaedic Surgery and Traumatology
- Canadian Orthopaedic Association
- European Federation of National Associations of Orthopaedics and Traumatology
- Mid-America Orthopaedic Association
- Piedmont Orthopedic Society
- Association of Bone and Joint Surgeons
- American Academy of Orthopaedic Surgeons
- Austrian Trauma Society
- Austrian Society for Orthopaedics and Orthopaedic Surgery
- German Society for Orthopaedics and Trauma,
- German Society for Orthopaedics, and Orthopaedic Surgery and the German Trauma Society

**Appendix 3**

**Effect of timing of surgery on mortality – forest plots for different cut-offs**

[Figure 5]

[Figure 6]

[Figure 7]

[Figure 8]

[Figure 9]

[Figure 10]

[Figure 11]
